# GSDMD contributes to host defence against *Staphylococcus aureus* skin infection by suppressing the Cxcl1–Cxcr2 axis

**DOI:** 10.1186/s13567-021-00937-7

**Published:** 2021-05-19

**Authors:** Zhen-Zhen Liu, Yong-Jun Yang, Feng-Hua Zhou, Ke Ma, Xiao-Qi Lin, Shi-Qing Yan, Yu Gao, Wei Chen

**Affiliations:** Key Laboratory of Zoonosis Research, Ministry of Education, College of Veterinary Medicine, Jilin University, Changchun, China

**Keywords:** *Staphylococcus aureus* (MRSA), Skin infection, GSDMD, Innate immune, Pyroptosis

## Abstract

Gasdermin D (GSDMD), a member of the gasdermin protein family, is a caspase substrate, and its cleavage is required for pyroptosis and IL-1β secretion. To date, the role and regulatory mechanism of GSDMD during cutaneous microbial infection remain unclear. Here, we showed that GSDMD protected against *Staphylococcus aureus* skin infection by suppressing Cxcl1–Cxcr2 signalling. GSDMD deficiency resulted in larger abscesses, more bacterial colonization, exacerbated skin damage, and increased inflammatory cell infiltration. Although GSDMD deficiency resulted in defective IL-1β production, the critical role of IL-1β was counteracted by the fact that Caspase-1/11 deficiency also resulted in less IL-1β production but did not aggravate disease severity during *S. aureus* skin infection. Interestingly, GSDMD-deficient mice had increased Cxcl1 secretion accompanied by increased recruitment of neutrophils, whereas Caspase-1/11-deficient mice presented similar levels of Cxcl1 and neutrophils as wild-type mice. Moreover, the absence of GSDMD promoted Cxcl1 secretion in bone marrow-derived macrophages induced by live, dead, or different strains of *S. aureus*. Corresponding to higher transcription and secretion of Cxcl1, enhanced NF-κB activation was shown in vitro and in vivo in the absence of GSDMD. Importantly, inhibiting the Cxcl1–Cxcr2 axis with a Cxcr2 inhibitor or anti-Cxcl1 blocking antibody rescued host defence defects in the GSDMD-deficient mice. Hence, these results revealed an important role of GSDMD in suppressing the Cxcl1–Cxcr2 axis to facilitate pathogen control and prevent tissue damage during cutaneous *S. aureus* infection.

**Supplementary Information:**

The online version contains supplementary material available at 10.1186/s13567-021-00937-7.

## Introduction

*Staphylococcus aureus* is highly pathogenic and can cause a wide range of clinical infections in both humans and domestic animals, leading to a major impact on public health and agriculture [1, 2]. The high burden of *S. aureus* among human and animal hosts is coupled with the well-known ability of this zoonotic pathogen to become resistant to antibiotics. Due to increasing drug resistance, current effective antibiotics continuously show decreased efficacy or even failure. Livestock-associated methicillin-resistant *S. aureus* is a serious problem worldwide. Some *S. aureus* strains have even developed resistance to the last-resort antibiotic vancomycin, and vaccine candidates have thus far been unsuccessful [3]. Further elucidation of the immune mechanisms of host resistance to *S. aureus* skin infection is needed to guide future host-directed therapies.

Innate immunity is involved in the early recognition and elimination of invading pathogens. Sensing pathogen-associated molecular patterns (PAMPs) through pattern recognition receptors (PRRs) enables host cells to categorize microbial invaders and to initiate appropriate host defence responses. Diverse cell types in the skin function as sentinel cells to recognize *S. aureus* components and contribute to cutaneous host defence, including TLRs 1, 2, and 6 in the cell membrane [4], TLR9 in endosomal membranes [5, 6], and STING [7] and NOD2 [8, 9] in the cytoplasm. An increased understanding of the innate immune mechanisms during *S. aureus* infection will help identify novel prevention and therapeutic strategies.

GSDMD is a member of the gasdermin family that consists of GSDMA, GSDMB, GSDMC, GSDMD, GSDME, and DFNB59. GSDMD was identified as the executioner of pyroptosis [10, 11], which is a type of programmed cell death different from apoptosis and necrosis [12]. A previously resolved crystal structure suggested that the C-terminal domain functions as an intrinsic inhibitor of GSDMD [13]. Once GSDMD is cleaved by inflammatory caspases, the N-terminal domain is liberated and can bind to biomembranes and mediate biomembrane perforation, followed by pyroptosis, the release of IL-1β and IL-18 and the loss of osmotic homeostasis [10, 14, 15]. GSDMD is involved in both cytokine release and cell death, indicating an important role of this molecule in controlling microbial infection. Studies have demonstrated that GSDMD plays a protective role in response to bacterial infections such as *Brucella abortus* [16], *Legionella pneumophila* [17], *Burkholderia thailandensis* [18], and *Francisella novicida* [19, 20]. However, much less is known about the biological implication of GSDMD in host defence during *S. aureus* skin infection.

In the current study, we demonstrated that GSDMD played a critical role in cutaneous defence against *S. aureus* infection. GSDMD deficiency resulted in increased Cxcl1 secretion in vivo and in vitro after *S. aureus* challenge. Inhibiting the Cxcl1–Cxcr2 axis rescued the defect of bacterial control in GSDMD^−/−^ mice. Collectively, our results suggested that GSDMD is essential for host defence against cutaneous *S. aureus* infection through inhibition of the Cxcl1–Cxcr2 axis.

## Materials and methods

### Mice

C57BL/6J wild-type (WT) mice were purchased from The Jackson Laboratory (Bar Harbor, ME, USA). GSDMD^−/−^ and Caspase-1/11^−/−^ mice were kindly provided by Prof. Feng Shao, National Institute of Biological Sciences, China [11]. These animals were subsequently backcrossed onto the C57BL/6J background for at least eight generations. Age and gender-matched WT controls were used. All animals were maintained on a standard 12/12-h light/dark cycle, fed standard rodent chow, provided water ad libitum, and housed in plastic cages with standard rodent bedding. The animal studies were conducted according to the experimental practices and standards approved by the Animal Welfare and Research Ethics Committee at Jilin University (No. 20150601).

### Model of skin infection

Mice were anaesthetized intraperitoneally (i.p.) with pentobarbital sodium (50 mg/kg) before shaving the dorsal area. The mice were then subcutaneously (s.c.) injected with the *S. aureus* strain USA300-TCH1516 (1 × 10^7^ CFU) in 50 μL of sterile phosphate buffered saline (PBS). The abscess area was measured using a Vernier caliper and photographed with a millimetre ruler as a reference. Mice were euthanized, and the lesional skin was collected by 8 mm punch biopsies at day 2 post-infection. A portion of the skin was homogenized mechanically in cold PBS (at a ratio of 5 mL per gram tissue), and the total bacterial burden was determined by plating out serial dilutions on tryptic soy broth (TSB) agar plates. In other experiments, 100 μg of SB225002 (Selleck, Houston, TX, USA) was administered i.p. 1 h prior to infection and 23 h post-infection. The control mice received equal amounts of vehicle. 5 μg of MAB453 (R&D, Emeryville, CA, USA) was administered i.p. 1 h prior to infection and 23 h post-infection. The control mice received equal amounts of isotype IgG.

### MPO assay

Skins collected from the WT and GSDMD^−/−^ mice after *S. aureus* infection were homogenized in 0.5% cetyltrimethylammonium chloride (5 µL/mg skin). The supernatants were collected for the MPO activity assay. Briefly, samples were transferred to a 96-well plate mixed with equal volumes (75 µL) of the substrate (3,3′, 5,5′ tetramethyl-benzidine dihydrochloride, 3 mmol/L; resorcinol, 6 mmol/L; and H_2_O_2_, 2.2 mmol/L) for 2 min. Then, 150 µL of 2 mol/L H_2_SO_4_ was used to stop the reaction. The OD was measured at 450 nm.

### Tissue histology and immunohistochemistry

Skin tissues were fixed in 4% paraformaldehyde and embedded in paraffin. For histology, skin sections (5 μm) were stained with haematoxylin and eosin (H&E). For immunohistochemistry, sections were deparaffinized, rehydrated, and subjected to antigen retrieval with citrate buffer, followed by blocking for 1 h at room temperature and incubation overnight at 4 ℃ with Ly-6G/Ly-6C (BioLegend, San Diego, CA, USA) and F4/80 (BioLegend) antibodies. After three washes with PBS, specific staining was detected using the UltraSensitive S-P Kit and DAB Detection Kit (Maixin-Bio, Fuzhou, China) according to the manufacturer’s directions. Sections were subsequently counterstained with haematoxylin, dehydrated, covered, and visualized under light microscopy.

### Cytokine and chemokine measurements

For measurement of the production of cytokines and chemokines in skin tissue, a part of the skin was homogenized mechanically in cold PBS (at a ratio of 5 mL per gram tissue) containing 1% Triton X-100 and a complete protease inhibitor cocktail (Sigma-Aldrich, St. Louis, MO, USA). Tissue lysates were then centrifuged at 12 000 *g* at 4 °C for 20 min, and the supernatants were then collected for later use. The ELISA kits were purchased from R&D Systems. Cytokines and chemokines in skin tissue or cell culture supernatants were measured by ELISAs according to the manufacturer’s instructions.

### Real-time PCR

RNA was isolated using TRI reagent (Sigma-Aldrich) and converted into cDNA. Subsequently, real-time PCR assays were performed using SYBR Green (Roche, Basel, Switzerland) on an ABI Prism 7500 sequence detection system (Life Tech [Applied BioSystems], Waltham, USA). Gene expression levels were calculated using the 2^−ΔCt^ method. The following primer sequences were used: GAPDH sense 5′-CACCCCAGCAAGG ACACTGAGCAAG-3′ and antisense 5′-G GGGGTCTGGGATGGAAATTGTGAG-3′. Cxcl1 sense 5′-TGAGCTGCGCTGTCAGTGCCT-3′ and antisense 5′-AGAAGCCAGCGTTCACCA GA-3′.

### Cell culture and infection

Bone marrow-derived macrophages (BMDMs) were obtained and cultured as previously described [21]. In short, bone marrow cells were sterilely isolated from femurs of 6- to 8-week-old mice and cultured in RPMI-1640 (Gibco, Waltham, MA, USA) containing 10% FBS (HyClone, Logan, UT, USA), 25% L929 cell-conditioned medium, 100 U/mL penicillin, and 100 U/mL streptomycin. On day 7 of differentiation, the cells were harvested for analysis. The BMDMs were seeded in 24-well cell culture plates containing 5 × 10^5^ cells or 6-well cell culture plates containing 3 × 10^6^ cells and cultured overnight. All *S. aureus* strains (USA300-TCH1516, 8325–4, and DU1090) were preserved in our laboratory. The BMDMs were treated with *S. aureus* (MOI 1:50) for 1 h, and the cells were washed twice with RPMI-1640 medium, cultured in RPMI-1640 medium containing 200 U/mL penicillin, 200 U/mL streptomycin, and 100 μg/mL gentamicin to remove excessive extracellular bacteria and then incubated at 37 ℃ for the designated time points.

### Western blotting

BMDMs or skins were harvested and then homogenized in basic RIPA buffer solution containing 1% Triton X-100, 50 mM Tris–HCl (pH 8.0), 150 mM NaCl, 0.25% sodium deoxycholate, and 0.1% SDS, and complete protease inhibitor cocktail (Sigma-Aldrich) was added. Total cell lysates were separated by SDS-PAGE and transferred to PVDF membranes. After the membranes were blocked with 5% milk, they were incubated with primary antibodies against p-P65 (Cell Signaling Technology, Beverly, MA, USA), p-IκBα (Cell Signaling Technology), IκBα (Cell Signaling Technology), and GAPDH (Proteintech, Chicago, IL, USA).

### Statistical analysis

Data are represented as the mean ± SEM. Data sets with only two independent groups were analysed for statistical significance using unpaired, two-tailed Student’s t-test. Data sets with more than two groups were analysed using one-way ANOVA with Tukey–Kramer post hoc tests. All *p* values less than 0.05 were considered significant (*p < 0.05, **p < 0.01, ***p < 0.001). Statistical analysis was performed using Prism (GraphPad Software, La Jolla, CA, USA).

## Results

### GSDMD contributes to host protection against *S. aureus* skin infection

**Figure 1:**
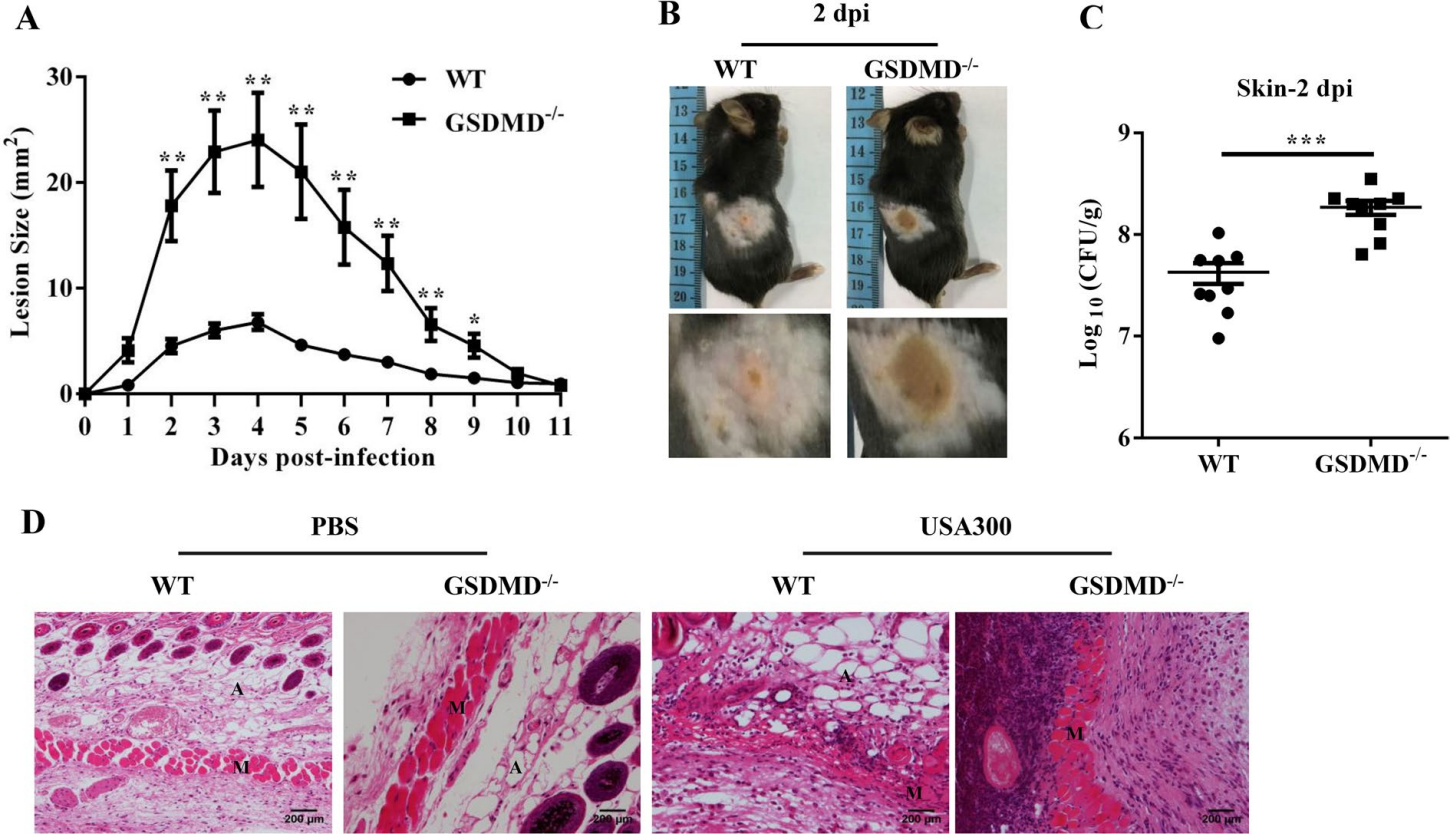
**GSDMD-deficient mice display increased susceptibility to** ***S. aureus*** **skin infection.** WT and GSDMD^−/−^ mice were infected s.c. with 1 × 10^7^ CFU *S. aureus.* **A** The lesion size from the dorsal area of mice from each group was measured. **B** Representative pictures are shown on day 2 post-infection. **C** Bacterial burden in the skin was assessed. **D** Skin tissue structures were observed by H&E (magnification of 200 ×). E: epidermis; D: dermis; A: adipose tissue; M: muscle fibres. All data are shown as the mean ± SEM. *n* = 9 per group. Data pooled from 2 independent experiments. Student’s *t*-test was performed. Statistical significance is indicated by *p < 0.05, **p < 0.01, and ***p < 0.001.

To evaluate the role of GSDMD in cutaneous host defence against *S. aureus* infection, we first compared the skin self-healing ability between the WT and GSDMD^−/−^ mice. We created cutaneous wounds (diameter = 0.5 cm) on the dorsal skin, and wound healing assays were performed. There was no significant difference between the two genotypes (Additional files 1A and B). Then, the WT and GSDMD^−/−^ mice were inoculated s.c. with 1 × 10^7^ CFU *S. aureus*. The abscess area was measured over an eleven-day infection period. The GSDMD^−/−^ mice formed significantly larger abscesses on days 2–9 than the WT mice (Figures 1A and B). The GSDMD^−/−^ mice had an increased bacterial burden in the skin compared to the WT mice on day 2 (Figure 1C) and on day 1 post-infection (Additional file 2). The abscess structure from the GSDMD^−/−^ mice was more severe than that from the WT mice on day 2 post-infection (Figure 1D). Collectively, these results suggested that GSDMD plays an important role in protection against *S. aureus* skin infection.

### GSDMD deficiency results in increased recruitment of inflammatory cells following *S. aureus* skin infection

**Figure 2:**
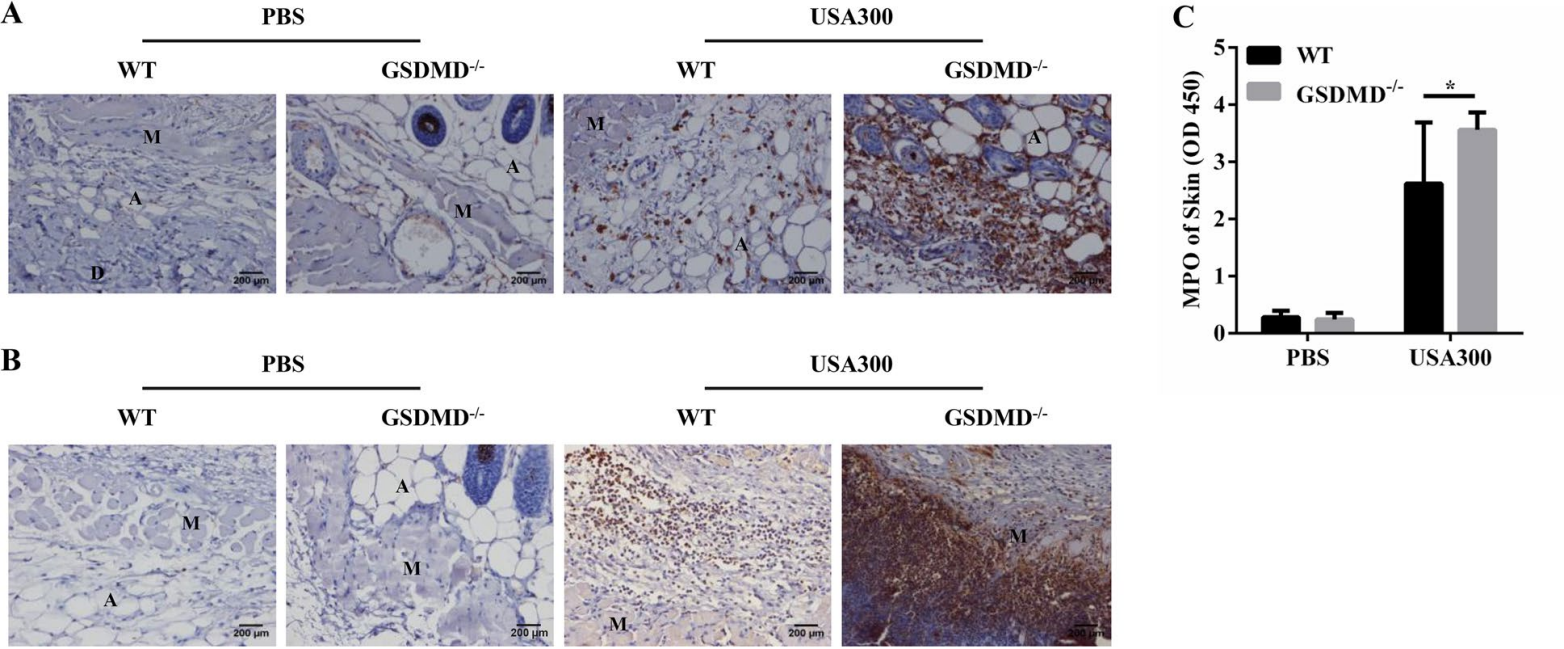
**GSDMD deficiency leads to enhanced inflammatory cell infiltration following** ***S. aureus* skin infection.** WT and GSDMD^−/−^ mice were infected s.c. with 1 × 10^7^ CFU *S. aureus,* and abscess tissue was excised on day 2 post-infection. Representative immunohistochemical staining of **A** F4/80 (a macrophagocyte marker) and **B** Gr-1 (a neutrophil marker) was performed in the skin sections. **C** The homogenate supernatants of skins were also used to determine the activity of MPO (a neutrophil marker). The data are shown as the mean ± SEM. *n* = 9 per group. Data were pooled from 2 independent experiments. Student’s *t*-test was performed. Statistical significance is indicated by *p < 0.05, **p < 0.01, and ***p < 0.001.

Infection and inflammation are always intertwined and are mutually a cause or consequence. Increased bacterial load aggravates inflammation, whereas excessive inflammation may exacerbate bacterial colonization. We next compared the recruitment of immune cells in the GSDMD^−/−^ and WT mice during *S. aureus* skin infection. By day 2, the GSDMD^−/−^ mice had significantly more macrophage and neutrophil infiltration than the WT mice at the site of infection (Figures 2A and B). As a neutrophil marker, the activity of myeloperoxidase (MPO) was higher in the GSDMD^−/−^ mice than in the WT mice (Figure 2C). Notably, the recruitment of neutrophils to the infected site is a hallmark of *S. aureus* infection [22]. Together, these results showed that GSDMD deficiency resulted in exaggerated recruitment of inflammatory cells that may cause tissue damage and promote infection during *S. aureus* skin infection.

### The GSDMD^−/−^ mice present decreased IL-1β and increased Cxcl1 secretion following *S. aureus* infection

**Figure 3:**
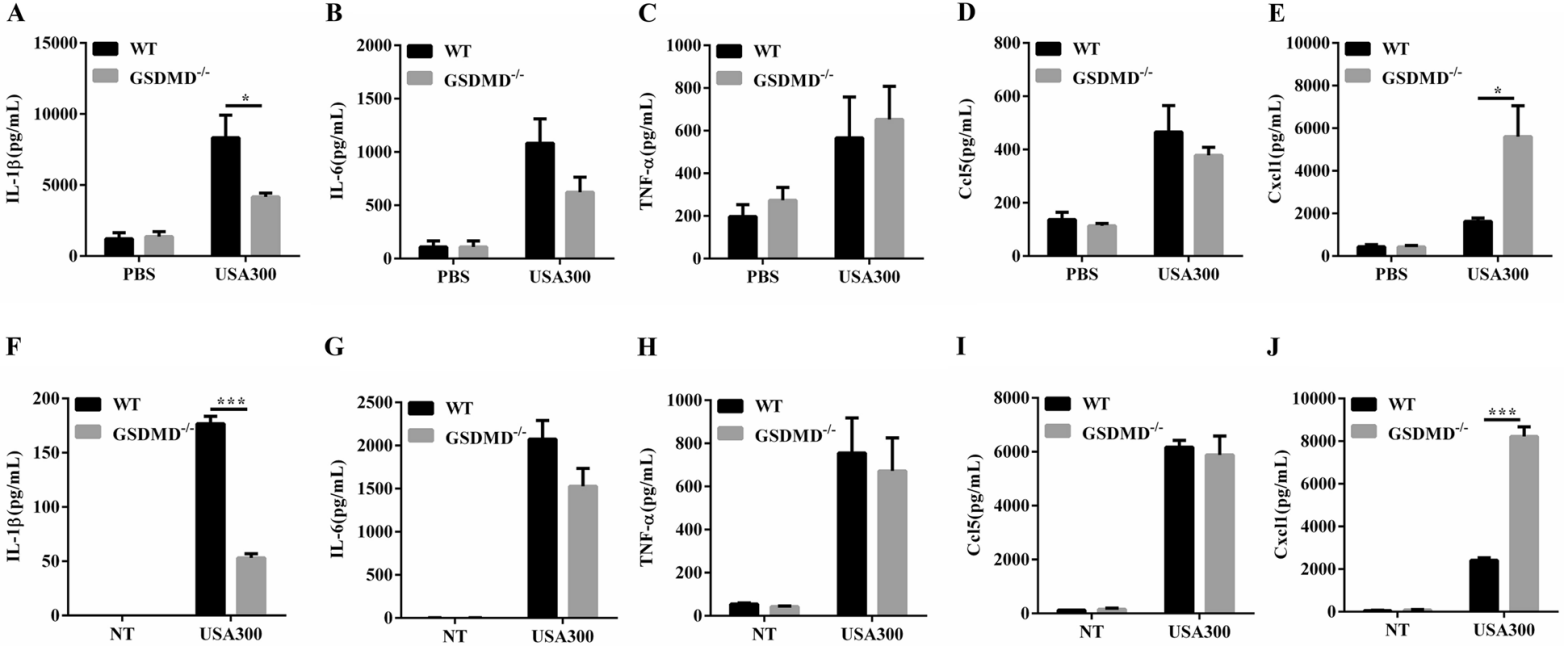
**Analysis of the role of GSDMD in producing cytokines/chemokines after *S. aureus* infection.** WT and GSDMD^−/−^ mice were infected s.c. with 1 × 10^7^ CFU *S. aureus,* and abscess tissue was excised on day 2 post-infection. The homogenate supernatants of skins were detected for concentrations of the indicated cytokines and chemokines by ELISAs. **A** IL-1β, **B** IL-6, **C** TNF-α, **D** Ccl5, **E** Cxcl1. WT and GSDMD^−/−^ BMDMs were treated with *S. aureus* for 24 h (MOI 1: 50). **F**–**J** Culture supernatants from BMDMs were analysed for IL-1β, IL-6, TNF-α, Ccl5, and Cxcl1 by ELISAs. **A**–**E** Data are shown as the mean ± SEM. *n* = 8 per group. Data were pooled from 2 independent experiments. F-J Data are shown as the mean ± SEM (three independent experiments) of each group (*n* = 3). Student’s *t*-test was performed. Statistical significance is indicated by *p < 0.05, **p < 0.01, and ***p < 0.001.

To further determine the skin inflammatory responses and given that cytokine/chemokine levels change substantially as a function of time and location during neutrophil recruitment [23, 24], we examined the secretion of inflammatory cytokines/chemokines at 1 and 2 days post-infection. The amounts of inflammatory cytokines (IL-1β) were lower in the skin of the GSDMD^−/−^ mice than in that of the WT mice at both time points (Figure 3A, Additional file 3A). Inflammatory cytokines (IL-6, TNF-α) and chemokine (Ccl5) were comparable between the genotypes (Figures 3B–D, Additional files 3B–D). Moreover, the chemokine Cxcl1 levels were significantly increased in the skin of the GSDMD^−/−^ mice at both time points (Figure 3E, Additional file 3E). Thus, it is logical to speculate that in the absence of GSDMD, either a decrease in IL-1β mediated weak protection or an increase in Cxcl1 exacerbated infection.

### Caspase-1/11 deficiency also results in decreased IL-1β production but does not aggravate skin infection

**Figure 4:**
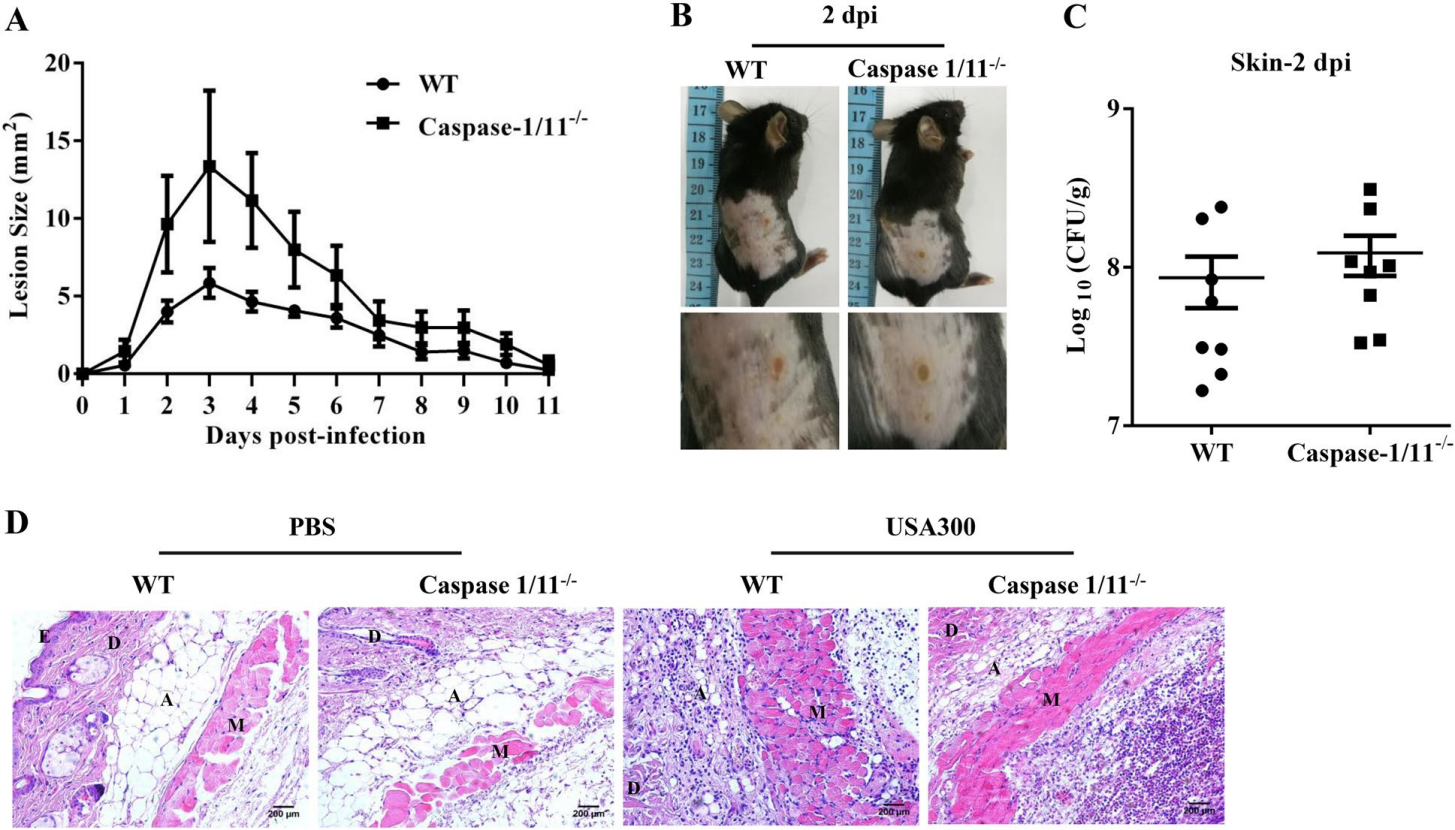
**Caspase-1/11 is dispensable for host defence against** ***S. aureus* skin infection.** WT and Caspase-1/11^−/−^ mice were infected s.c. with 1 × 10^7^ CFU *S. aureus.* **A** The lesion size from the dorsal area of mice from each group was measured. **B** Representative pictures are shown on day 2 post-infection. **C** Bacterial burden in the skin was assessed. **D** Skin tissue structures were observed by H&E (magnification of 200 ×). E: epidermis; D: dermis; A: adipose tissue; M: muscle fibres. All data are shown as the mean ± SEM. Student’s *t*-test was performed. *n* = 8 per group. Data were pooled from 2 independent experiments. Statistical significance is indicated by *p < 0.05, **p < 0.01, and ***p < 0.001.

Caspase-1/11 specifically cleaves the linker domain of GSDMD and allows the amino-terminal domain to oligomerize and form pores on the plasma membrane of cells. The pore-forming activity of GSDMD-N is required for cell death and facilitates the release of IL-1β. To examine whether decreased IL-1β confers weak protection in the absence of GSDMD, we used Caspase-1/11^−/−^ mice. Although the Caspase-1/11^−/−^ mice exhibited slightly increased lesions compared to the WT mice, there was no significant difference between the two genotypes (Figures 4A and B). The bacterial burden (Figure 4C) and abscess structure (Figure 4D) of both genotypes were not different. However, compared with those in the WT mice, the amounts of IL-1β were lower in the skin of the Caspase-1/11^−/−^ mice, similar to the GSDMD^−/−^ mice (Additional file 4A). Consistent with the lack of a significant difference in the infection phenotype between the WT and Caspase-1/11^−/−^ mice, the levels of the chemokine Cxcl1 were also comparable (Additional file 4B). Collectively, the infection outcomes of mice of the three genotypes were not consistent with the IL-1β expression levels but consistent with the neutrophil chemoattractant Cxcl1. Although neutrophils are critical for the clearance of pathogens, excessive recruitment can induce substantial tissue/organ damage [25]. We speculated that the increase in Cxcl1 levels rather than the decrease in IL-1β levels may account for the more severe infection in the GSDMD^−/−^ mice. We focused on Cxcl1 to clarify whether it could be a key gene promoting the inflammatory reaction in the GSDMD^−/−^ mice.

### GSDMD deficiency promotes* S. aureus*-induced Cxcl1 production in a NF-κB-dependent manner

**Figure 5:**
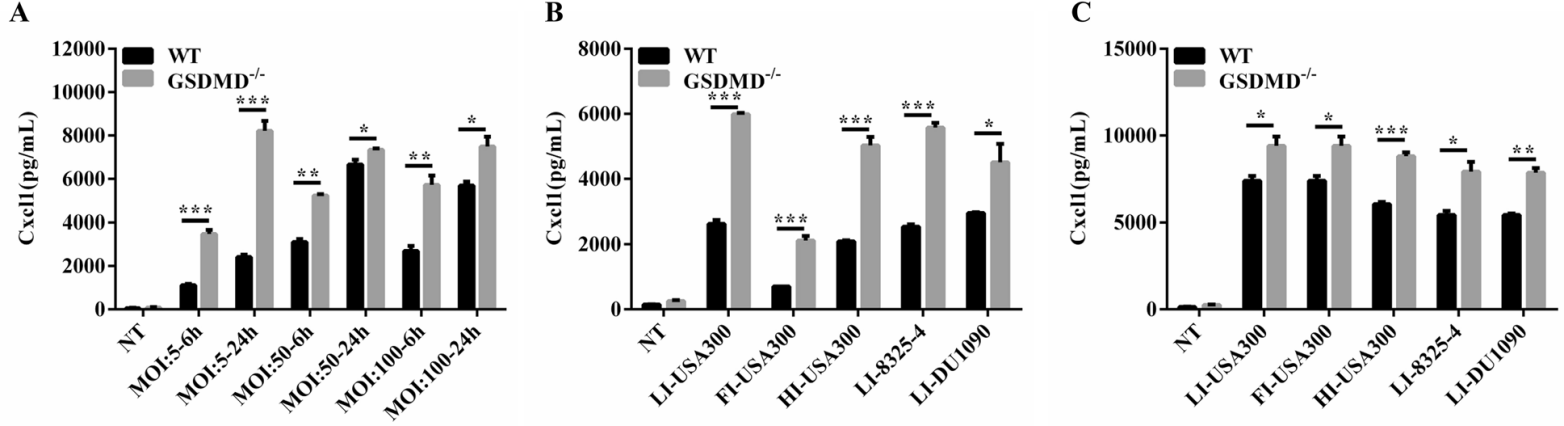
**GSDMD inhibits Cxcl1 secretion in bone marrow-derived macrophages. A** WT and GSDMD^−/−^ BMDMs were treated with *S. aureus* for 6 h and 24 h (MOI 1: 5, 1: 50, and 1: 100). Culture supernatants from BMDMs were analysed for Cxcl1 by ELISAs. WT and GSDMD^−/−^ BMDMs were treated with *S. aureus* (USA300, 8325–4, and DU1090) for 6 h **B** and 24 h **C** (MOI 1: 100). Culture supernatants from BMDMs were analysed for Cxcl1 by ELISAs. LI: live; FI: formaldehyde-inactivated; HI: heat-inactivated. Data are shown as the mean ± SEM (three independent experiments) of each group (*n* = 3). Student’s *t*-test was performed. Statistical significance is indicated by *p < 0.05, **p < 0.01, and ***p < 0.001.

**Figure 6:**
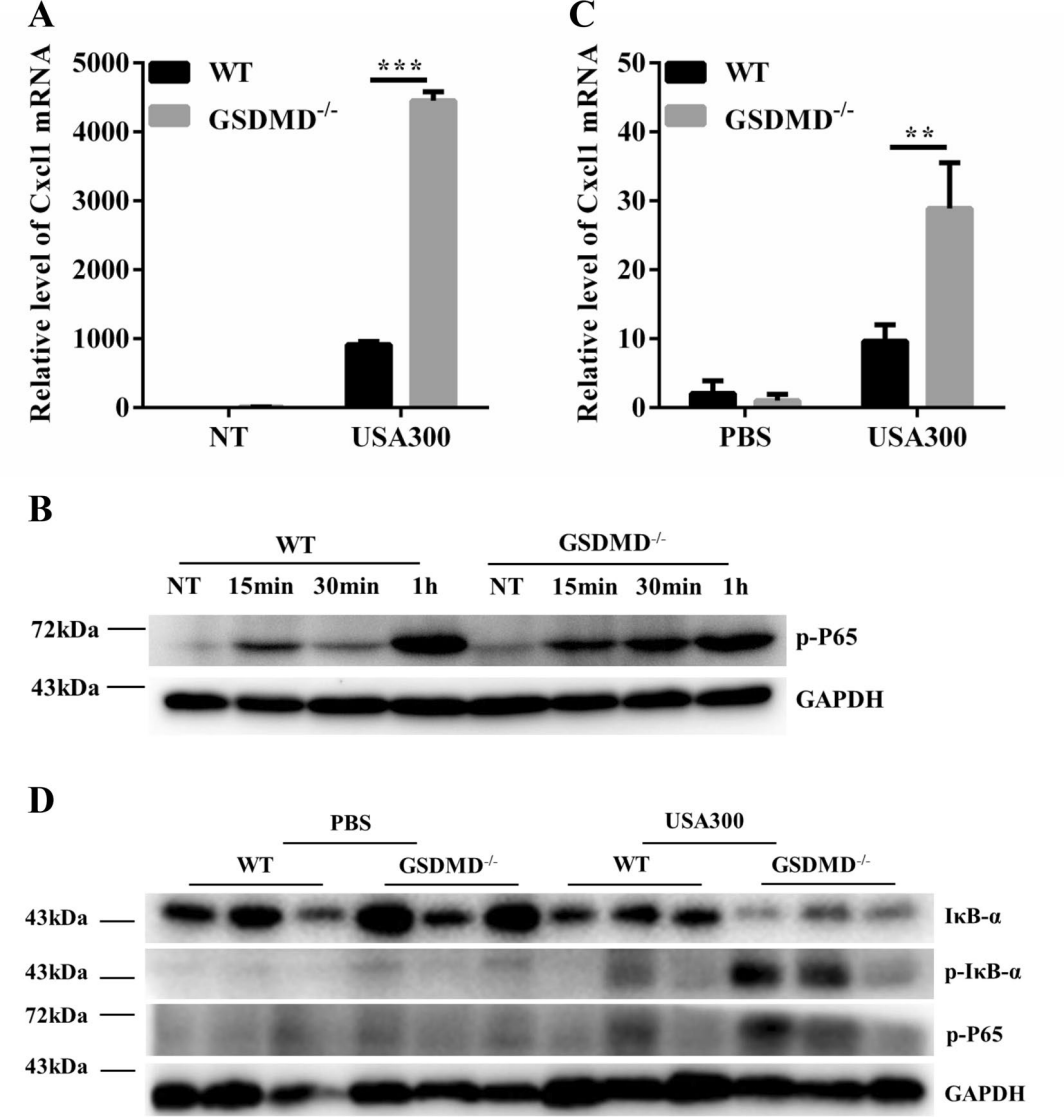
**GSDMD inhibits Cxcl1 secretion in a NF-κB-dependent manner upon** ***S. aureus* challenge.** WT and GSDMD^−/−^ BMDMs were untreated or exposed to *S. aureus* at an MOI of 1:50 for the indicated times. **A** Cxcl1 mRNA levels were measured at 6 h post-infection by qRT-PCR. **B** The cell lysates were examined for the expression levels of p-P65 by Western blotting. GAPDH served as a loading control. WT and GSDMD^−/−^ mice were infected s.c. with 1 × 10^7^ CFU *S. aureus,* and abscess tissue was excised on day 2 post-infection. **C** Skin tissue mRNA was examined for Cxcl1 by qRT-PCR. **D** Skin tissues were homogenized and then immunoblotted for p-P65, p-IκBα, IκBα, and GAPDH. Data A are shown as the mean ± SEM (three independent experiments) of each group (*n* = 3). Data C are shown as the mean ± SEM. *n* = 8 per group. Data were pooled from 2 independent experiments. Student’s *t*-test was performed. Statistical significance is indicated by *p < 0.05, **p < 0.01, and ***p < 0.001.

GSDMD was mainly expressed in inflammatory cells in the skin (data not shown). Moreover, tissue macrophages are the main source of Cxcl1 in the skin [26]. BMDMs derived from the WT and GSDMD^−/−^ mice were infected with *S. aureus* at MOIs of 1:5, 1:50, and 1:100, and the secretion level of Cxcl1 was examined. Cxcl1 levels were significantly increased in the GSDMD^−/−^ BMDMs at both the 6- and 24-h time points compared to those of the WT BMDMs (Figure 5A). In addition, heat- or formaldehyde-inactivated *S. aureus* (USA300) and other *S. aureus* strain 8325-4 or Hla-deficient strain DU1090 were used to infect the BMDMs. Compared with those of the WT BMDMs, high levels of Cxcl1 were found in the GSDMD^−/−^ BMDMs with different treatments at both the 6- and 24-h time points (Figures 5B and C). These results indicated that GSDMD deficiency promotes *S. aureus*-induced Cxcl1 secretion in macrophages, consistent with the in vivo data. BMDMs isolated from the WT and GSDMD^−/−^ mice were infected with *S. aureus*, and the protein expression levels of inflammatory cytokines and chemokines were detected and were consistent with the results in vivo (Figures 3F–J). We further characterized the mRNA expression of Cxcl1 in macrophages and skin. Cxcl1 mRNA expression was upregulated in the absence of GSDMD in vivo and in vitro (Figures 6A and C). NF-κB, a transcription factor, is involved in the expression of genes related to the inflammatory process, including regulated Cxcl1 expression. NF-κB signalling was largely enhanced in the absence of GSDMD, as shown by the increased phosphorylation level of p65 in the GSDMD^−/−^ BMDMs (Figure 6B). Consistently, the skin tissues of the GSDMD^−/−^ mice challenged with bacteria showed a similar trend of significantly increased NF-κB activation, as demonstrated by the increased phosphorylation levels of p65 and IκBα compared with those of the WT mice (Figure 6D). Thus, these results suggested that GSDMD deficiency promotes *S. aureus*-induced Cxcl1 production in a NF-κB-dependent manner.

### Inhibition of the Cxcl1–Cxcr2 axis attenuates the enhanced skin infection in the GSDMD^−/−^ mice

**Figure 7:**
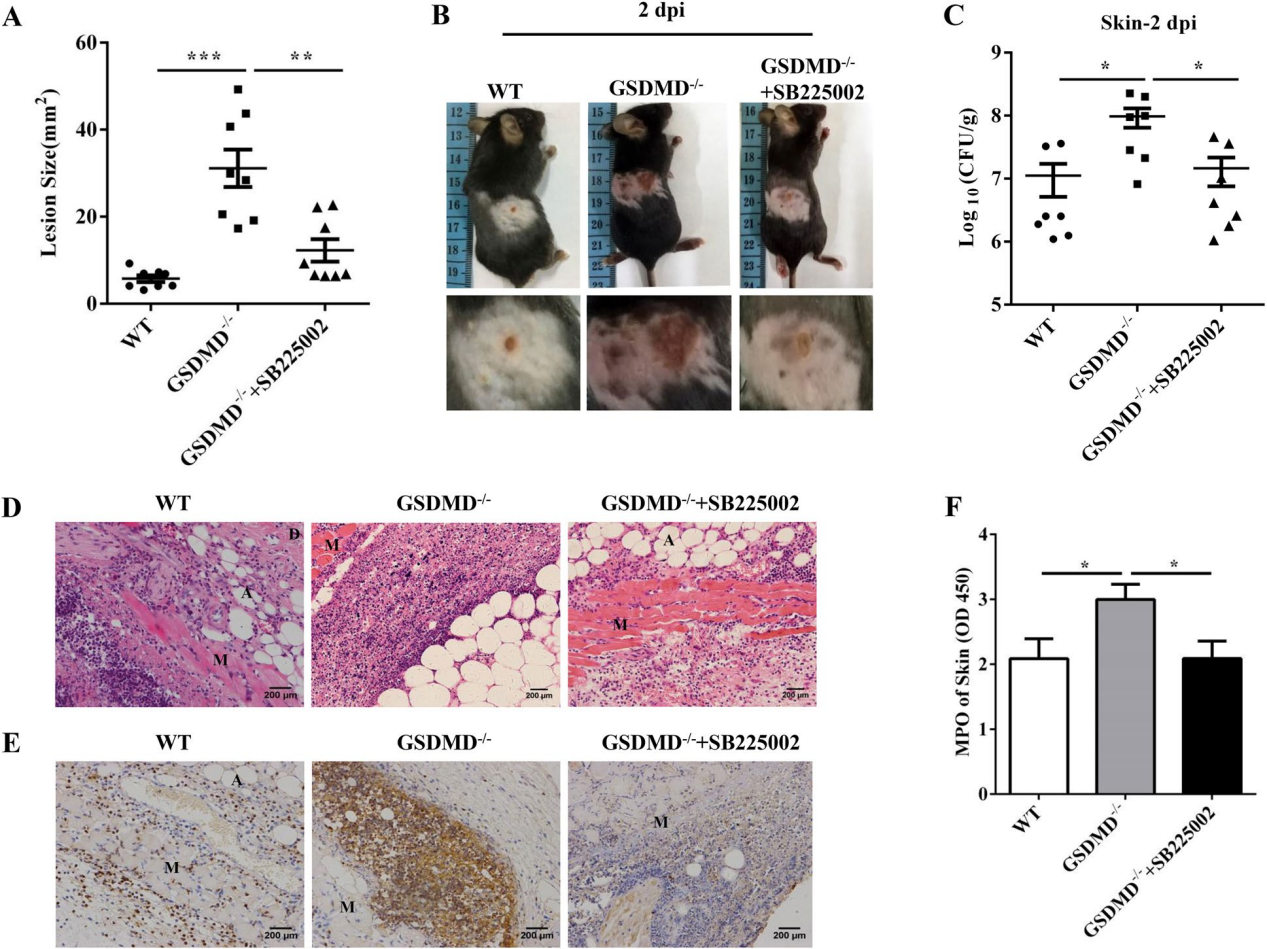
**Inhibiting the Cxcl1–Cxcr2 axis in the GSDMD**^**−/−**^** mice improves host defence during** ***S. aureus* skin infection.** For one group of GSDMD^−/−^ mice, 100 μL of 100 μg SB225002 was injected i.p. 1 h prior to infection and 23 h post-infection with 1 × 10^7^ CFU *S. aureus*, and abscess tissue was excised on day 2 post-infection. **A** The lesion size from the dorsal area of mice from each group was measured. **B** Representative pictures are shown. **C** Bacterial burden in the skin was assessed. **D** Skin tissue structures were observed by H&E (magnification of 200 ×). **E** Representative immunohistochemical staining of Gr-1 was performed in the skin sections. **F** The homogenate supernatants of skins were also used to determine the activity of MPO. E: epidermis; D: dermis; A: adipose tissue; M: muscle fibres. All data are shown as the mean ± SEM. *n* = 7–8 per group. Data pooled from 2 independent experiments. One-way ANOVA with Tukey–Kramer post hoc tests was performed. Statistical significance is indicated by *p < 0.05, **p < 0.01, and ***p < 0.001.

Cxcl1 is the main Cxcr2 ligand required for neutrophil transendothelial migration. Next, we evaluated whether inhibiting the Cxcl1–Cxcr2 axis in the GSDMD^−/−^ mice could rescue impaired host defence against *S. aureus* skin infection. The GSDMD^−/−^ mice were treated i.p. with SB225002 (Cxcr2 inhibitor) or vehicle 1 h prior to infection and 23 h post-infection. The GSDMD^−/−^ mice treated with SB225002 exhibited decreased abscesses and presented comparable levels to those of the WT mice (Figures 7A and B). Moreover, SB225002 treatment resulted in a reduced bacterial burden in the skin of the GSDMD^−/−^ mice, indicating that inhibiting the Cxcl1–Cxcr2 axis benefited bacterial control in the GSDMD^−/−^ mice during cutaneous *S. aureus* infection (Figure 7C). The abscess structure was dramatically attenuated in the GSDMD^−/−^ mice treated with SB225002, nearly to the extent observed in the WT mice after *S. aureus* skin infection (Figure 7D). Moreover, inhibiting the Cxcl1–Cxcr2 axis decreased the accumulation of neutrophils (Figures 7E and F). Collectively, these results showed that the Cxcl1–Cxcr2 axis is responsible for the decreased host defence against *S. aureus* skin infection in the GSDMD^−/−^ mice.

### Blocking Cxcl1 in GSDMD^−/−^ mice augments host defence against* S. aureus* skin infection

**Figure 8:**
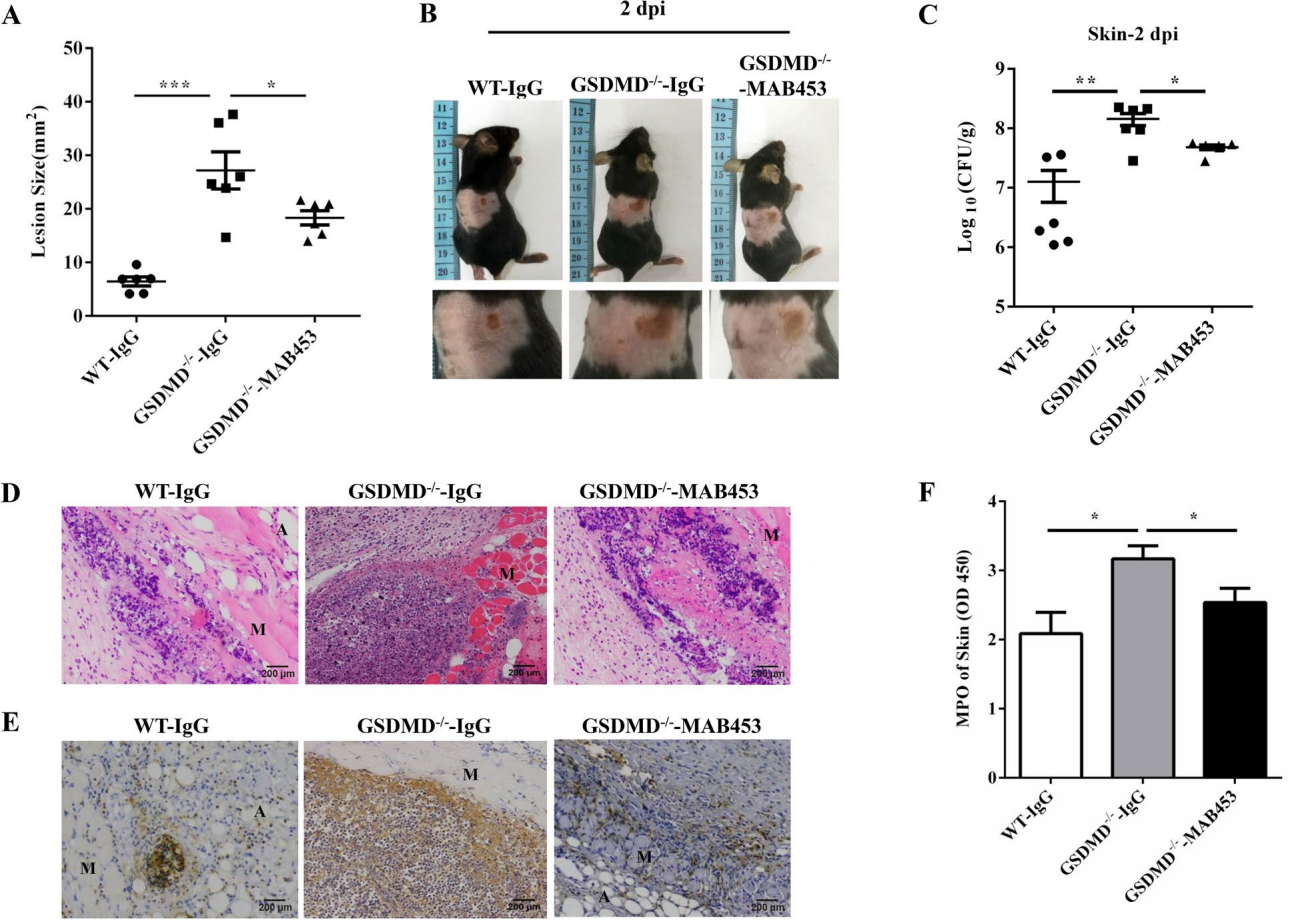
**Blocking Cxcl1 in the GSDMD**^**−/−**^** mice improves host defence during** ***S. aureus* skin infection.** For one group of GSDMD^−/−^ mice, 100 μL of 5 μg MAB453 was injected i.p. 1 h prior to infection and 23 h post-infection with 1 × 10^7^ CFU *S. aureus*, and abscess tissue was excised on day 2 post-infection. **A** The lesion size from the dorsal area of mice from each group was measured. **B** Representative pictures are shown. **C** Bacterial burden in the skin was assessed. **D** Skin tissue structures were observed by H&E (magnification of 200 ×). **E** Representative immunohistochemical staining of Gr-1 was performed in the skin sections. **F** The homogenate supernatants of skins were also used to determine the activity of MPO. E: epidermis; D: dermis; A: adipose tissue; M: muscle fibres. All data are shown as the mean ± SEM. *n* = 6 per group. Data pooled from 2 independent experiments. One-way ANOVA with Tukey–Kramer post hoc tests was performed. Statistical significance is indicated by *p < 0.05, **p < 0.01, and ***p < 0.001.

The pivotal role of Cxcl1 was further confirmed when it was neutralized by the anti-Cxcl1 blocking antibody MAB453. The GSDMD^−/−^ mice were treated i.p. with MAB453 or IgG 1 h prior to infection and 23 h post-infection. The GSDMD^−/−^ mice treated with MAB453 exhibited decreased abscesses (Figures 8A and B). Moreover, MAB453 treatment resulted in a reduced bacterial burden in the skin of the GSDMD^−/−^ mice (Figure 8C). The abscess structure was dramatically attenuated in the GSDMD^−/−^ mice treated with MAB453 (Figure 8D). Moreover, blocking Cxcl1 decreased the accumulation of neutrophils (Figures 8E and F). These findings suggested that GSDMD facilitates pathogen control and prevents tissue damage via Cxcl1 suppression during cutaneous *S. aureus* infection.

## Discussion

*S. aureus* skin infections can often cause life-threatening infections, including pneumonia, meningitis, septic arthritis, endocarditis, and sepsis [27]. Although antibiotics can restrict bacterial growth and proliferation, the formation of cutaneous abscesses could limit the penetration and efficacy of antibiotics [28]. In addition, there is currently no approved vaccine available for *S. aureus* treatment. Therefore, a detailed understanding of the host innate immune response is crucial for the discovery of potential novel therapeutic targets. Pyroptosis has emerged as a key mechanism in antimicrobial innate immune defence [12]. The protein GSDMD is the executor of pyroptosis and can be cleaved by inflammatory caspases [11, 29]. Previous studies demonstrated that GSDMD is highly expressed in the epithelial and cutaneous systems [30]. However, the biological implications of GSDMD in bacterial infections have not been characterized.

In our study, we revealed a novel mechanism by which GSDMD protected against *S. aureus* skin infection by suppressing Cxcl1–Cxcr2 signalling. We first demonstrated that the GSDMD^−/−^ mice were more susceptible to *S. aureus* infection than the WT mice, which was manifested by significantly larger abscesses, a greater bacterial burden in the skin, severe destruction of the skin architecture, and increased inflammatory cell infiltration. We examined the secretion of inflammatory cytokines/chemokines that may account for the immune response. The GSDMD^−/−^ mice presented decreased IL-1β and increased Cxcl1 secretion following *S. aureus* infection, suggesting that upon *S. aureus* challenge, promotion of IL-1β production or inhibition of Cxcl1 may be the key for the protective effect of GSDMD during bacterial infection.

Inflammasomes are formed in response to invasive microbial pathogens or signs of intracellular danger. The canonical inflammasome is an intracellular multimolecular complex that forms a platform to activate caspase-1 and in turn cleaves pro-IL-1β, pro-IL-18, and GSDMD, which induces GSDMD to form pores in the plasma membrane and promotes pyroptosis [31]. Previous studies have clearly shown that the NLRP3 inflammasome is critical for promoting IL-1β activity by inducing excessive neutrophil recruitment to the site of infection in the skin [32, 33]. Subsequent studies noted that ASC-deficient mice have a similar phenotype as IL-1β-deficient mice, mainly reflected in the larger lesions, increased bacterial counts, and defective neutrophil recruitment compared with WT mice after *S. aureus* cutaneous challenge [33]. We hypothesized that defects in IL-1β production may lead to susceptibility to infection in the absence of GSDMD. The contribution of IL-1β to host defence against *S. aureus* cutaneous infection was tested in the Caspase-1/11^−/−^ mice. Unexpectedly, the Caspase-1/11^−/−^ mice exhibited no significant difference in abscesses, bacterial burden in the skin, or abscess structure compared with the WT mice. This finding differed from that in the study by Kitur et al., who reported that Caspase-1/11 activity is beneficial for *S. aureus* clearance and that Caspase-1/11^−/−^ mice had increased Cxcl1 levels and slightly, but not significantly, lower IL-1β levels post-infection [34]. Importantly, our study revealed that the Caspase-1/11^−/−^ mice presented decreased levels of IL-1β and comparable levels of Cxcl1 compared to the WT mice. The difference may be related to the difference in the virulence of bacterial strains, infectious dose, and measured endpoints. Hence, IL-1β was not the key factor for the protective effect of GSDMD in our study.

Neutrophils are involved in innate immune defence against invading pathogens through multiple mechanisms, including phagocytosis, reactive oxygen species (ROS), antimicrobial peptides, enzymatic digestion, and proteins that sequester essential nutrients, as well as via the formation of neutrophil extracellular traps (NETs). Patients with congenital or acquired defects in neutrophil number or function were reported to be highly susceptible to *S. aureus* skin infections [35], whereas the inappropriate accumulation and activation of neutrophils can augment unwanted tissue damage [36]. Multiple factors contribute to neutrophil recruitment from the circulation to the site of infection, including proinflammatory cytokines (such as IL-1α, IL-1β, TNF-α, and IL-6) and Cxcr2 chemokines (such as Cxcl1, Cxcl2, Cxcl5, and Cxcl8). Cxcr2^−/−^ mice show impaired neutrophil recruitment and delayed wound healing [37, 38]. Thus, we speculated that increased Cxcl1 secretion might account for the excessive neutrophil recruitment in the GSDMD^−/−^ mice. Tissue macrophages are the first line of defence against pathogens when microorganisms invade and are the main source of neutrophil-attracting chemokines in the skin [26, 39]. The number of tissue macrophages is small, and macrophages are difficult to sort by flow cytometry. Thus, BMDMs are usually used for further in vitro experiments. Herein, our results showed that expression of the chemokine Cxcl1 was significantly increased in vivo and in vitro in the GSDMD^−/−^ mice. Consistent with previous studies, a failure to induce Cxcl1 production was associated with a defect in neutrophil infiltration in inflamed skin [40]. Moreover, the absence of GSDMD promoted Cxcl1 secretion in BMDMs induced by live, dead, or different strains of *S. aureus*.

NF-κB is a key transcription factor that controls the expression of multiple proinflammatory cytokines and chemokines, including Cxcl1 [41]. NF-κB has been found to regulate Cxcl1 transcription during *S. pneumoniae* and *Pseudomonas aeruginosa* infection [42, 43]. In our study, enhanced NF-κB activation corresponded to higher transcription and secretion of Cxcl1 in vitro and in vivo in the absence of GSDMD. More importantly, inhibiting the Cxcl1–Cxcr2 axis with a Cxcr2 inhibitor or anti-Cxcl1 blocking antibody rescued defects in host defence against *S. aureus* skin infection in the GSDMD^−/−^ mice. To date, few studies on GSDMD associated with chemokines have been reported. Li et al. found that GSDMD-mediated hepatocyte pyroptosis expands the inflammatory response by upregulating Ccl2/Ccr2 expression to recruit macrophages [44]. In contrast to their reports, we demonstrated a previously unknown regulatory role for GSDMD in inhibiting the Cxcl1–Cxcr2 signalling response to *S. aureus* infection. GSDMD-mediated inhibition of inflammatory signalling is not uncommon. Similarly, Banerjee et al. showed that GSDMD-driven K^+^ efflux could inhibit cGAS-dependent type I interferon production [19]. Therefore, we hypothesize that GSDMD may share a related mechanism in maintaining ionic homeostasis that restrains the NF-κB-Cxcl1–Cxcr2 axis, which requires further study.

In conclusion, the present study reveals the beneficial role of GSDMD during *S. aureus* skin infection. GSDMD promotes bacterial killing and prevents excess tissue damage during *S. aureus* skin infection. However, IL-1β does not contribute to GSDMD-mediated protection. Moreover, GSDMD inhibits the Cxcl1–Cxcr2 axis with a key role in neutrophil recruitment. These observations document a novel and physiologically important role for GSDMD in host defence against *S. aureus* infection. A better understanding of the gasdermin family may provide new targets for effective treatment for infectious diseases that impact human health.

## Supplementary Information

**Additional file 1. GSDMD does not affect the self-healing ability of skin.** Cutaneous wounds (diameter = 0.5 cm) were generated on the dorsal skin of the WT and GSDMD^−/−^ mice, and wound healing assays were performed. (A) Representative pictures are shown on days 0, 1, 2, and 4. (B) The lesion size from the dorsal area of mice from each group was measured. All data are shown as the mean ± SEM. *n* = 6 per group. Data were pooled from 2 independent experiments. Student’s *t*-test was performed. Statistical significance is indicated by *p < 0.05, **p < 0.01, and ***p < 0.001.

**Additional file 2. GSDMD facilitates pathogen control during cutaneous** ***S. aureus*** **infection.** WT and GSDMD^−/−^ mice were infected s.c. with 1 × 10^7^ CFU *S. aureus,* and abscess tissue was excised on day 1 post-infection. Bacterial burden in the skin was assessed. Data are shown as the mean ± SEM. *n* = 8–9 per group. Data were pooled from 2 independent experiments. Student’s *t*-test was performed. Statistical significance is indicated by *p < 0.05, **p < 0.01, and ***p < 0.001.

**Additional file 3. Analysing the role of GSDMD in producing cytokines/chemokines after** ***S. aureus*** **infection. WT and GSDMD**^**−/−**^ **mice were infected s.c. with 1 × 10**^**7**^** CFU** ***S. aureus*, and abscess tissue was excised on day 1 post-infection.** The homogenate supernatants of skins were detected for concentrations of the indicated cytokines and chemokines by ELISA. (A) IL-1β, (B) IL-6, (C) TNF-α, (D) Ccl5, (E) Cxcl1. Data are shown as the mean ± SEM. *n* = 8 per group. Data were pooled from 2 independent experiments. Student’s *t*-test was performed. Statistical significance is indicated by *p < 0.05, **p < 0.01, and ***p < 0.001.

**Additional file 4. The secretion of IL-1β and Cxcl1 in the WT, Caspase-1/11**^**−/−**^**, and GSDMD**^**−/−**^ **mice after** ***S. aureus*** **skin infection.** WT, Caspase-1/11^−/−^**,** and GSDMD^−/−^ mice were infected s.c. with 1 × 10^7^ CFU *S. aureus,* and abscess tissue was excised on day 1 post-infection. The homogenate supernatants of skins were detected for concentrations of (A) IL-1β and (B) Cxcl1. All data are shown as the mean ± SEM. *n* = 6 per group. Data were pooled from 2 independent experiments. One-way ANOVA with Tukey–Kramer post hoc tests was performed. Statistical significance is indicated by *p < 0.05, **p < 0.01, and ***p < 0.001.

## Data Availability

All data generated or analysed during this study are included in this published article.

## Abbreviations

*S. aureus*: *Staphylococcus aureus*
GSDMD: Gasdermin D
CA-MRSA: Community-acquired methicillin-resistant *Staphylococcus aureus*
PAMPs: Pathogen-associated molecular patterns
PRRs: Pattern recognition receptors
TLR: Toll-like receptor
NOD2: Nod-like receptor 2
STING: Stimulator of interferon genes
s.c.: Subcutaneously
i.p.: Intraperitoneally
PBS: Phosphate buffered saline
TSB: Tryptic soy broth
MOI: Multiplicity of infection
BMDM: Bone marrow-derived macrophage
CFU: Colony-forming unit
H&E: Haematoxylin and eosin
MPO: Myeloperoxidase
