# Supplementary figures and images for: GSDMD contributes to host defence against Staphylococcus aureus skin infection by suppressing the Cxcl1–Cxcr2 axis
Source: Vet Res. 2021 May 19;52:71. doi: 10.1186/s13567-021-00937-7 (PMC8132424; doi:10.1186/s13567-021-00937-7)

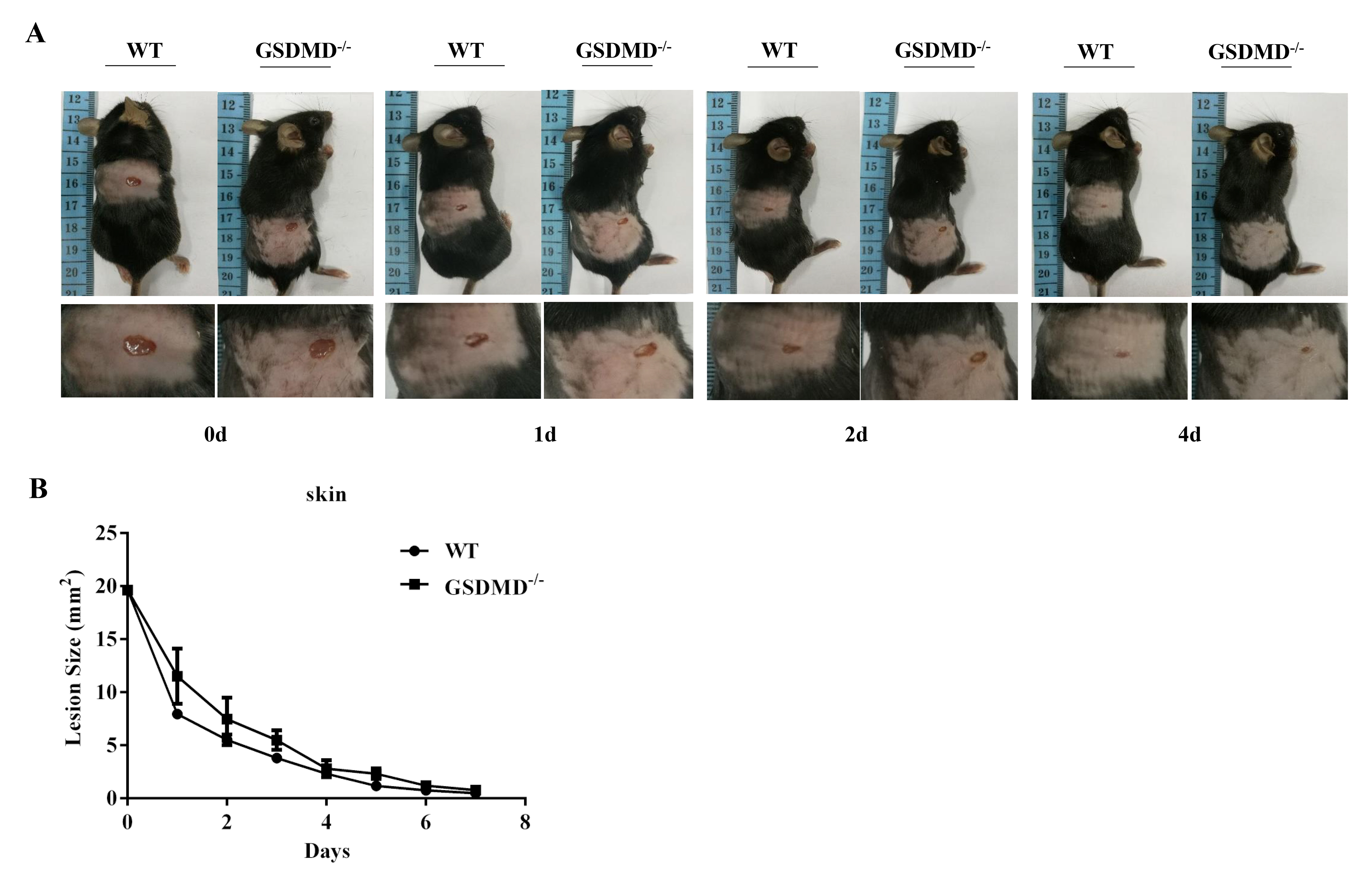

Supplement: Supplementary file 1 — Additional file 1. GSDMD does not affect the self-healing ability of skin. Cutaneous wounds (diameter = 0.5 cm) were generated on the dorsal skin of the WT and GSDMD−/− mice, and wound healing assays were performed. (A) Representative pictures are shown on days 0, 1, 2, and 4. (B) The lesion size from the dorsal area of mice from each group was measured. All data are shown as the mean ± SEM. n = 6 per group. Data were pooled from 2 independent experiments. Student’s t-test was performed. Statistical significance is indicated by *p < 0.05, **p < 0.01, and ***p < 0.001. [file 13567_2021_937_MOESM1_ESM.jpg]

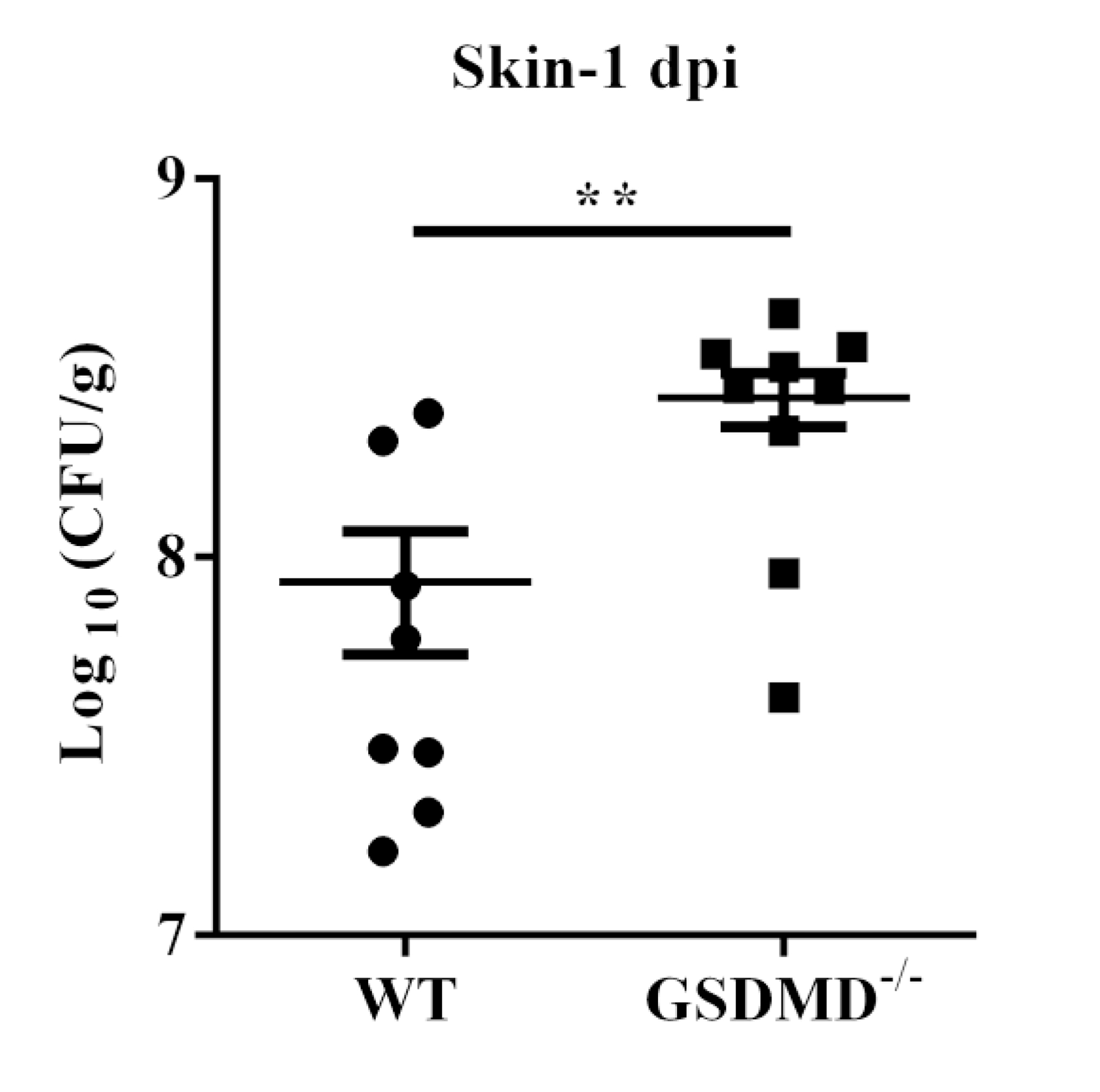

Supplement: Supplementary file 2 — Additional file 2. GSDMD facilitates pathogen control during cutaneous S. aureus infection. WT and GSDMD−/− mice were infected s.c. with 1 × 10^7^ CFU S. aureus, and abscess tissue was excised on day 1 post-infection. Bacterial burden in the skin was assessed. Data are shown as the mean ± SEM. n = 8–9 per group. Data were pooled from 2 independent experiments. Student’s t-test was performed. Statistical significance is indicated by *p < 0.05, **p < 0.01, and ***p < 0.001. [file 13567_2021_937_MOESM2_ESM.jpg]

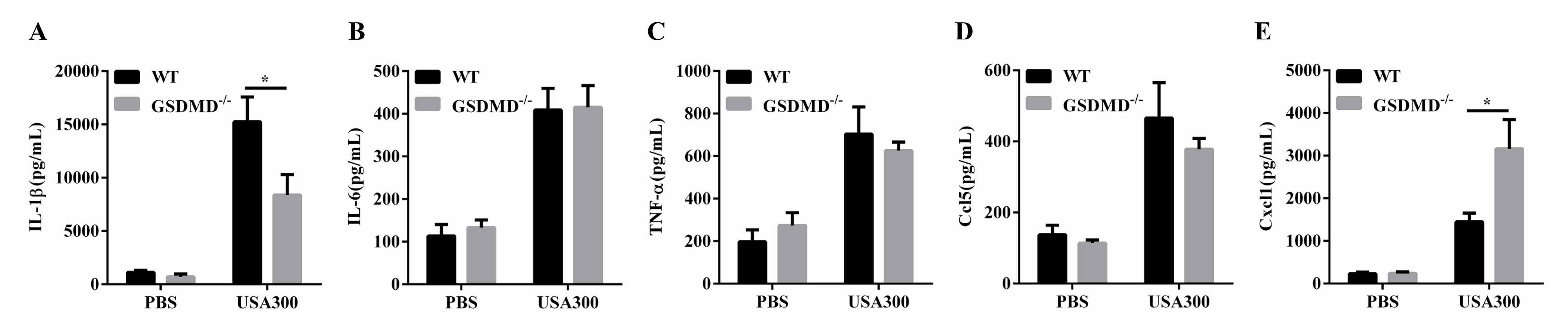

Supplement: Supplementary file 3 — Additional file 3. Analysing the role of GSDMD in producing cytokines/chemokines after S. aureus infection. WT and GSDMD−/− mice were infected s.c. with 1 × 10^7^ CFU S. aureus, and abscess tissue was excised on day 1 post-infection. The homogenate supernatants of skins were detected for concentrations of the indicated cytokines and chemokines by ELISA. (A) IL-1β, (B) IL-6, (C) TNF-α, (D) Ccl5, (E) Cxcl1. Data are shown as the mean ± SEM. n = 8 per group. Data were pooled from 2 independent experiments. Student’s t-test was performed. Statistical significance is indicated by *p < 0.05, **p < 0.01, and ***p < 0.001. [file 13567_2021_937_MOESM3_ESM.jpg]

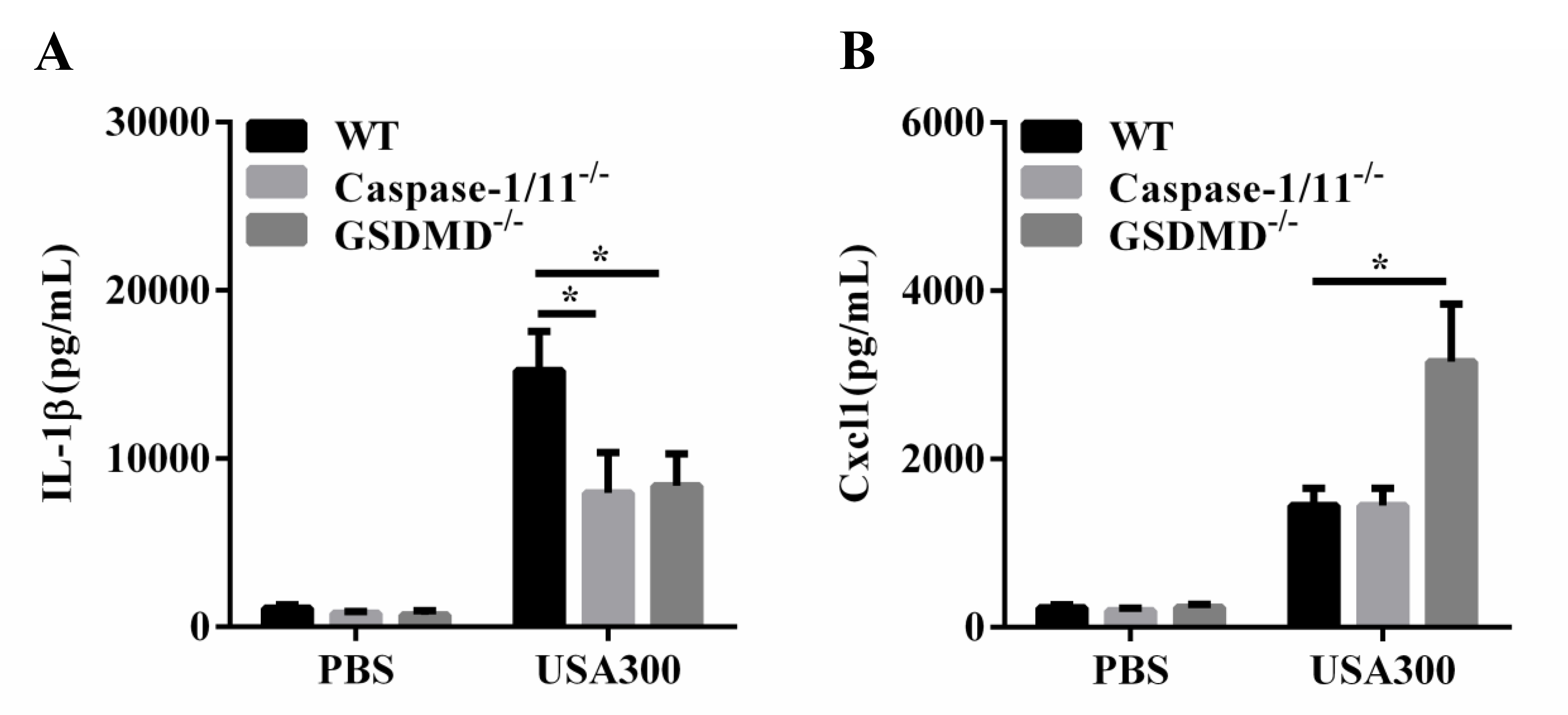

Supplement: Supplementary file 4 — Additional file 4. The secretion of IL-1β and Cxcl1 in the WT, Caspase-1/11−/−, and GSDMD−/− mice after S. aureus skin infection. WT, Caspase-1/11−/−, and GSDMD−/− mice were infected s.c. with 1 × 10^7^ CFU S. aureus, and abscess tissue was excised on day 1 post-infection. The homogenate supernatants of skins were detected for concentrations of (A) IL-1β and (B) Cxcl1. All data are shown as the mean ± SEM. n = 6 per group. Data were pooled from 2 independent experiments. One-way ANOVA with Tukey–Kramer post hoc tests was performed. Statistical significance is indicated by *p < 0.05, **p < 0.01, and ***p < 0.001. [file 13567_2021_937_MOESM4_ESM.jpg]
